# Pulse length, energy spread, and temporal evolution of electron pulses generated with an ultrafast beam blanker

**DOI:** 10.1063/1.5089517

**Published:** 2019-04-26

**Authors:** I. G. C. Weppelman, R. J. Moerland, L. Zhang, E. Kieft, P. Kruit, J. P. Hoogenboom

**Affiliations:** 1 Imaging Physics, Delft University of Technology, Lorentzweg 1, 2628 CJ Delft, The Netherlands; 2 School of Instrumentation Science and Opto-electronics Engineering, Beihang University, 100191 Beijing, China; 3 Thermo Fisher Scientific, Achtseweg Noord 5, 5651 GG Eindhoven, The Netherlands

## Abstract

Crucial for the field of ultrafast electron microscopy is the creation of sub-picosecond, high brightness electron pulses. The use of a blanker to chop the beam that originates from a high brightness Schottky source may provide an attractive alternative to direct pulsed laser illumination of the source. We have recently presented the concept of a laser-triggered ultrafast beam blanker and argued that generation of 100 fs pulses could be possible [Weppelman *et al.*, Ultramicroscopy **184**, 8–17 (2017)]. However, a detailed analysis of the influence of a deflection field changing sign on sub-picoseconds time scale on the quality of the resulting electron pulses has so far been lacking. Here, we present such an analysis using time-dependent, three-dimensional numerical simulations to evaluate the time-evolution of deflection fields in and around a micrometers-scale deflector connected to a photo-conductive switch. Further particle tracing through the time-dependent fields allows us to evaluate beam quality parameters such as energy spread and temporal broadening. We show that with a shielded, “tunnel-type” design of the beam blanker limiting the spatial extent of fringe fields outside the blanker, the blanker-induced energy spread can be limited to 0.5 eV. Moreover, our results confirm that it could be possible to bring laser-triggered 100 fs focused electron pulses on the sample using a miniaturized ultrafast beam blanker. This would enable us to resolve ultrafast dynamics using focused electron pulses in an SEM or STEM.

## INTRODUCTION

Ultrafast Electron Microscopy (UEM) is an emerging field of research where the aim is to image structural dynamics at ultrafast time scales with high spatial resolution. Both imaging and diffraction modes, as well as Electron Energy Loss Spectroscopy (EELS), can be used in UEM to study ultrafast dynamics in materials.^2–4^ UEM systems have also enabled the study of quantum mechanical interactions between photons and electrons.^5^ For all these applications, it is important to generate high brightness electron pulses as the brightness directly determines the amount of current that can be used to illuminate the sample with a particular beam opening angle. High brightness ultrafast electron pulses, with peak brightness comparable to Schottky emitters, can be created using laser pulse illumination of a sharp metal tip^6^ or alternatively by using a microwave cavity to chop a continuous electron beam.^7^ The latter method has been implemented in a Transmission Electron Microscope (TEM), and both numerical calculations and experimental measurements have shown the conservation of beam emittance.^8,9^ Both laser source illumination and insertion of a microwave cavity may require extensive modification of the electron microscope column.

We have recently presented an alternative concept for obtaining short, focused electron pulses, which relied on the integration of an electrostatic beam blanker with a photoconductive switch in a single device made with MEMS technology.^1,10^ Such an ultrafast blanker (UFB) could be inserted into the column of an existing electron microscope, thus allowing us to rapidly alternate between static DC operation and time-resolved imaging using electron pulses. Based on back-of-the-envelope calculations, simplified models, and reported experimental performance of photoconductive Auston switches, we argued that with a MEMS-sized UFB it should be possible to create electron pulses of about 100 fs focused in a 10 nm spot at an acceleration voltage of 30 kV. These pulses would then contain on average about 0.5 electrons per pulse in order to maintain the beam quality between blanker and sample. However, we also calculated that the time-response of an UFB would be critically influenced by the capacitance of the deflector electrode.^1^ This capacitance is difficult to estimate with analytical equations and challenging to measure experimentally. Also, it is difficult to incorporate the full response of a photoconductive switch and, thus, the temporal dynamics of the blanking voltage in an analytical model, while this may influence the achievable brightness (or emittance) and the energy spread in the pulse. Both brightness and energy spread are important to evaluate as brightness determines the obtainable current, and the energy spread is important for the reason that chromatic aberrations cause a decrease in spatial resolution and limit the spectral resolution in EELS measurements. For UEM, energy spread is also important as it causes a temporal broadening when the pulse travels from blanker to the sample.

Fowler and Good have shown that in general creating electron pulses by chopping a continuous beam may reduce the beam quality.^11^ Equations describing the energy spread induced by a blanker have been derived by Thong for different blanker configurations, such as conjugate blanking and sweeping a focused beam over a blanking aperture.^12^ Thong concluded that, to first order, a conjugate blanker does not introduce any additional energy spread. Oldfield analyzed the beam quality for a combination of two cavities where the first is used for chopping the beam and the second for correcting the induced energy spread.^13^ Further analyses of conjugate beam blanking using magnetic deflection fields inside a resonant radio frequency field (RF) cavity have been performed by Lassise *et al.*^8,14,15^ They showed that their RF blanker will introduce negligible energy spreads for 100 fs electron pulses at 30 keV beam energies. This system has now been incorporated in a TEM.^9^ A MEMS-sized electrostatic beam blanker driven by sinusoidal RF fields has been analyzed by Cook. He found that such a blanker introduced a negligible increase in emittance and energy spread apart from a 1.7 eV constant energy gain for 400 fs electron pulses.^16^ Our MEMS-sized UFB controlled by a photoconductive switch is significantly smaller and uses a broadband deflection field with frequencies up to the terahertz range. Recently, experiments have been performed demonstrating the possibility of terahertz fields to control electron pulses and to measure terahertz fields by, respectively, Kealhofer *et al.* and Ryabov *et al.*^17,18^ The potential emittance growth and energy spread introduced by such deflection fields in a MEMS device have so far not been addressed. In this manuscript, we present time-dependent, three-dimensional numerical simulations to better evaluate the time-dependent deflection fields inside the UFB and, thus, the influence of the UFB on the quality of the electron pulses. In order to perform such simulations, a model of the time-dependent conductivity of an Auston switch is required, which is also discussed below. This model can then be used to calculate the response of the photoconductive switch under illumination of a laser pulse and, in combination with Maxwell's equations, be used to numerically evaluate the time-dependent deflection fields in and around the UFB.

## SIMULATION SETUP, MODEL, AND APPROXIMATIONS

**FIG. 1.**
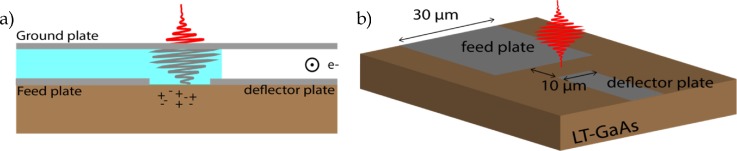
Sketch of UFB with a photoswitch located between the feed and deflector plate of the blanker. The electron beam propagates between the ground plate and deflector plate perpendicular to this plane as indicated. A glass layer indicated in light blue separates the ground plate from the feed plate. (b) Side view of (a) with the ground plate removed and typical dimensions indicated.

First, we briefly review the UFB concept, which has been described in full detail elsewhere.^10^ The beam blanker consist of two plates, one grounded and the other connected to a photoconductive switch as indicated in Fig. 1, called the deflector plate. The other electrode of the photoconductive switch is connected to an electrical circuit delivering a voltage, called the feed plate. The beam blanker is integrated with a photoswitch in a single micrometers scaled device. The photoconductive switch is activated by a femtosecond laser pulse that creates free carriers in the semiconductor material, resulting in a current due to the bias field over the switch. This current is used to (de)charge the deflector plate of the beam blanker; hence, in a short time scale the deflection field in the blanker will be inverted. A DC electron beam propagating between the blanker plates will sweep over an aperture and an ultrafast electron pulse is created below this blanking aperture. Numerical simulations are conducted using Comsol Multiphysics modeling software.

**FIG. 2.**
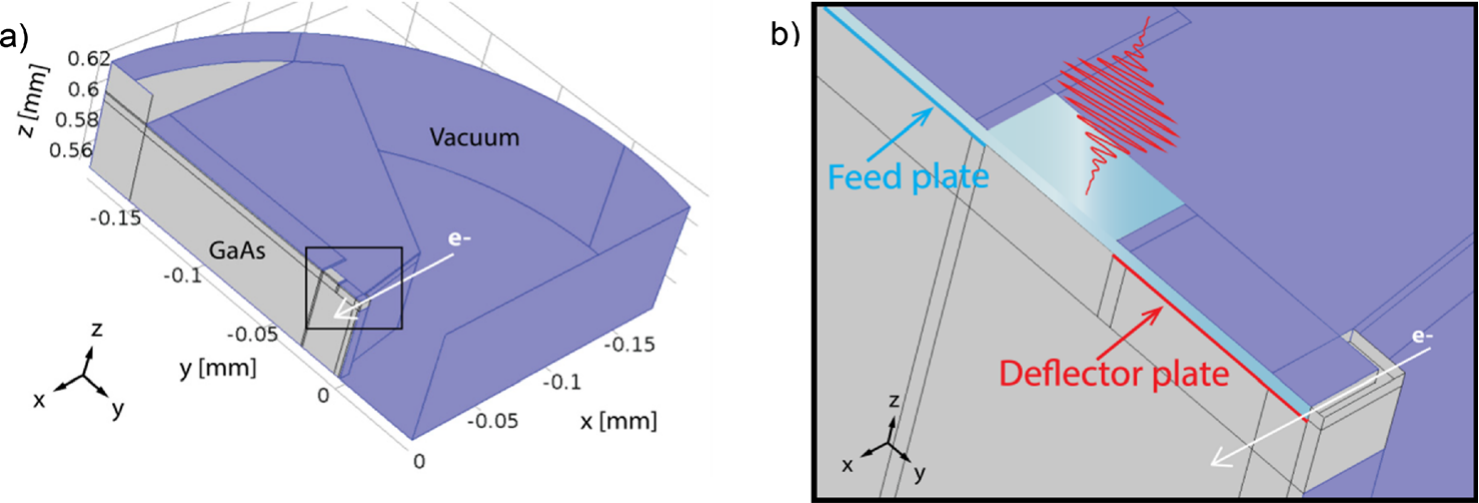
UFB simulation geometry. (a) A half pillbox is used, the fields are symmetrical at the flat (left) side, and the electrons will elastically reflect from the symmetry boundary. All blue plates are electrically grounded perfect metals and, hence, are reflecting boundaries for the time-dependent simulation. The white arrow shows the propagation direction of the electron beam along the x-direction. (b) Zoom in at the position of the photo switch and blanker [indicated with a black square in (a)]. The deflector plate is indicated with a red line and the feed plate with a blue line. The electron beam is deflected due to the voltage difference between the deflector plate and ground. In light blue, the glass layer is indicated which separates the ground plates and the electrodes, and an opening in the ground plate is present such that the laser pulse can illuminate the photoconductive switch.

Figure 2 shows how the UFB design was implemented in Comsol Multiphysics, where we used a half symmetry to reduce the calculation time and memory space required.

The simulation of the UFB and photoconductive switch consists of three steps. First, we calculate the electrostatic potential in the whole domain. A Poisson solver is used to calculate the electrostatic potential, and the photoconductive switch is assumed to be a perfect electrical insulator. The voltage of the feed plate and the deflector plate is −10 V and +10 V, respectively. A relative permittivity of 12.25 is assumed for GaAs and a permittivity of 2.25 is used for the SiO_2_ layer that separates the ground plate and deflector/feed plates.

The calculated surface charge density on the deflector plate is used to determine the capacitance of the deflector plate. We found that a 15 *μ*m long deflector plate results in a desired capacitance of 6.6 fF: a factor of 2 higher than expected according to our previous back-of-the-envelope calculation shown in Weppelman *et al.*,^1^ which assumed a parallel plate capacitor. We attribute this to the fact that the high permittivity GaAs supporting the capacitor is not included in the parallel plate capacitor model. Also, parasitic capacitances and the complicated geometry around the deflector were not taken into account in the simplified model.

The calculated potential is then used as an initial condition in the second step for the full (time-dependent) transient Maxwell solver. Hereto, a time-dependent conductivity is required for the photoconductive switch. In the section on “Model for the time-dependent,” we explain the calculation of the conductivity of the photoconductive switch. Then, the last step is to trace electrons through the time-dependent electromagnetic fields.

## MODEL FOR THE TIME-DEPENDENT CONDUCTIVITY OF THE PHOTOCONDUCTIVE SWITCH

The laser illumination creates free carriers (electrons and holes) in the photoconductive switch which are subsequently accelerated due to the electric field applied over the switch. In the model, we only consider free electrons, which have a dominant contribution to conductivity. The reason is that the effective mass of electrons is an order of magnitude lower than of holes in GaAs.^19^ The relation between the conductivity, electric field, and current density is linear,

j=σE. (1)

The current density is equal to

j=n(t)e⟨v(t)⟩, (2)

where *n*(*t*) is the density of free carriers as function of time, generated by the laser pulse and *⟨v⟩* is the average drift velocity of free electrons.

The average drift velocity is described in each mesh point by the following equation based on a Drude-Lorentz model:

$$\frac{d\left\langle v\right\rangle }{\mathrm{dt}}=-\frac{\left\langle v\right\rangle }{\tau_{s}}+\frac{e}{m*}\left|E\left(t\right)\right|s\mathrm{ign}(E_{y}\left(t\right))-\left\langle v\right\rangle \frac{G(x,t)}{n(x,t)},$$ (3)

where *E*(*t*) is the local electric field and *E_y_* is the field in *y-*direction as defined in Fig. 2, *n*(***x***, *t*) is the density of free carriers as function of time and space, *G*(***x***, *t*) is the time derivative of *n*(***x***, *t*), i.e., the generation rate of new free electrons. The scattering time, *τ_s_*, is estimated to be 30 fs and the effective mass, m*, is equal to 0.067 times the rest mass of an electron.^20^ Drude-Lorentz models are often successfully used in the literature to describe the dynamics of charges in Auston switches, for example, by Jepsen *et al.*,^20^ Piao *et al.*,^21^ and Duvillaret *et al.*^22^

Free electrons will be displaced over a typical distance of 100 nm in a time scale of 1 ps, based on a maximum drift velocity of 1 × 10^5^ m/s which we also used in our previous work.^1^ Spatial diffusion can be neglected in Eq. (3) because the displacement is smaller than both the mesh size and electromagnetic wavelengths. In the numerical calculation, screening of the electric field in the photoconductive switch due to charge separation and coulomb interactions between the holes and electrons is neglected. According to the literature, this is a reasonable assumption as long as the carrier density is below 10^18^ cm^−3^.^20,21^

The density of free carriers, *n*(***x***, *t*), follows the time integral of the laser pulse, *G*(***x***, *t*), which has a Gaussian temporal envelope and is equal to

$$n\left(x,t\right)=\frac{1}{2}n_{t}\left(1+\text{erf}\left(t/1.67\tau \right)\right)g(x,y,z),$$ (4)

where *n_t_* is the total density of generated free carriers, *g*(*x*, *y*, *z*) is the spatial variation of the laser intensity, and τ the FWHM of the laser pulse. Recombination of electron-hole pairs is not taken into account because the recombination time is on the order of 10 to 15 ps, much longer than the time scales relevant in the simulation.

**FIG. 3.**
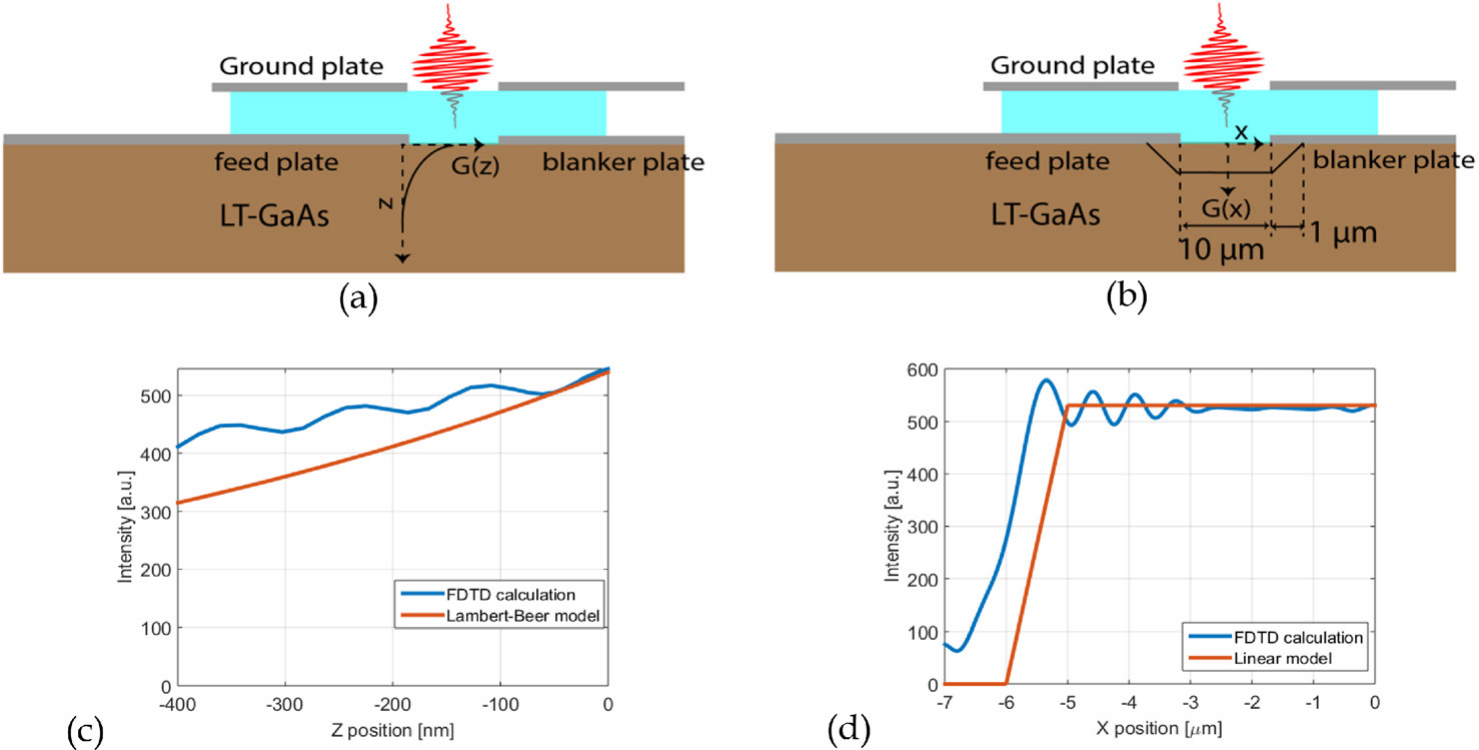
In our computational model, we use (a) an exponential decay of the intensity of the laser in the z-direction according to Lambert-Beer, and (b) a linear variation of the laser intensity in the y-direction. (c) FDTD calculation (blue line) of the laser intensity as function of z position in the GaAs at the center of the photoconductive switch. The glass-GaAs interface is located at z = 0. The red line is the laser intensity used in the calculation in Comsol. (d) FDTD calculation of the laser intensity as function of x at a depth of 10 nm below the glass-GaAs interface. The red line is the laser intensity assumed in the Comsol calculation. A broadband p-polarized plane wave located above the ground plate is injected in the FDTD simulation, and the resulting laser intensity is integrated over a range from 750 to 850 nm.

The *z*-dependence of the function *g*(x, y, z) in Eq. (4) takes into account that deeper into the GaAs less charges are generated due to the absorption of light in GaAs, as described by the Lambert–Beer model

$$I(z)=I_{0}\;\text{exp}\;\left(-4\pi \mathrm{kz}/\lambda \right)\approx I_{0}\;\text{exp}\;\left(-z/\lambda \right),$$ (5)

where *k* is the imaginary part of the index of refraction, in case of GaAs equal to 0.089 at 800 nm.^23^ Underneath the electrodes, the laser intensity is lower, and we use a rough approximation by simply assuming a homogenous laser intensity illuminating the photoconductive switch and linear decreasing field under the metal, as depicted in Fig. 3. To verify the linear model, we compared it with the laser intensity calculated with Lumerical, a commercial finite difference time domain (FDTD) solver of Maxwell's equations. In this simulation, a plane wave is injected just above the ground plate propagating toward the GaAs layer. The contact layers are modeled as molybdenum plates with a thickness of 30 nm, and the optical constants are taken from Ordal *et al.*^24^ As depicted in Fig. 3(b), the FDTD calculation confirms that the linear model of the laser intensity will give conservative estimations of the photoconductivity around the contact electrodes. Note that a very basic design for the electrodes is assumed for the calculation. More advanced electrodes with significant improvements in terms of photocurrent have been demonstrated in the literature; see, for example, the review paper of Lepehov *et al.* and references therin.^25^

The conductivity can be calculated by solving the ordinary differential Eq. (3),

σ=|⟨v⟩|ne/|E|. (6)

Note that the conductivity is dependent on the field and, in turn, the field is dependent on the conductivity. Therefore, the conductivity and Maxwell's equations are solved in an iterative loop, which does increase the CPU resources required for the simulation.

The calculation time can be reduced by assuming that the electric field over the switch is only slowly varying in time compared to the time it takes for the electrons to accelerate to the drift velocity. In that case, the conductivity of the photoconductive switch can be modeled as the convolution of the laser pulse and the velocity response of the carriers based on a Drude-Lorentz model assuming a constant electric field

$$\sigma (t)=\frac{\mathrm{en}(t)v(t)}{E}=\frac{e}{E}\int_{-\infty }^{\infty }G(t-t^{\prime })v(t^{\prime })dt^{\prime }=\frac{e^{2}}{m*}\int_{-\infty }^{\infty }\left(1-e^{-\frac{t^{\prime }}{\tau_{s}}}\right)G(t-t^{\prime })dt^{\prime }.$$ (7)

**FIG. 4.**
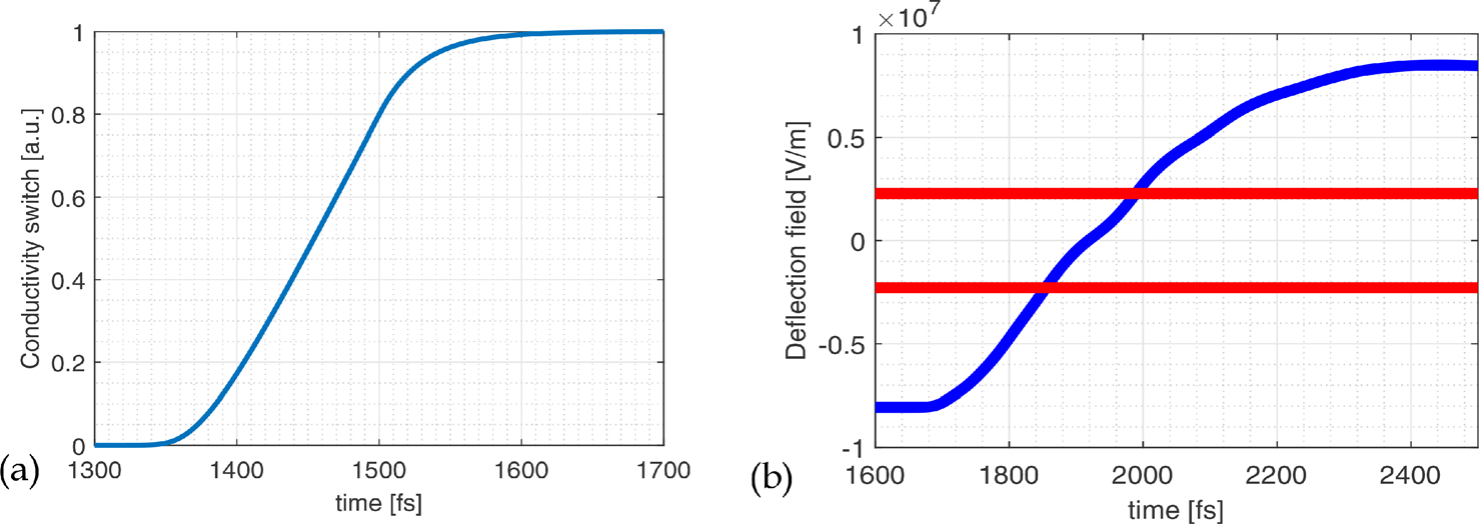
(a) Conductivity of the photoconductive switch vs time calculated using Eq. (7) assuming a 25 fs Gaussian short laser pulse. The conductivity is used to calculate (b) the deflection field in the blanker as a function of time. In the area between the red lines, the electron beam can partially pass the blanking aperture that accepts a half opening angle of 0.4 mrad.

The model of the time-dependent conductivity of the Auston switch, Eq. (7), is used to calculate the time-dependent electric field in the blanker, as shown in Fig. 4(b). The electric field between the two deflector plates inverts on a time scale of about 500 fs. As shown in Fig. 4, a time delay of about 200–400 fs is present between the conductivity built-up and the moment when the deflection field starts to change. This is caused by propagation delays over the 15 *μ*m long deflector plate.

**FIG. 5.**
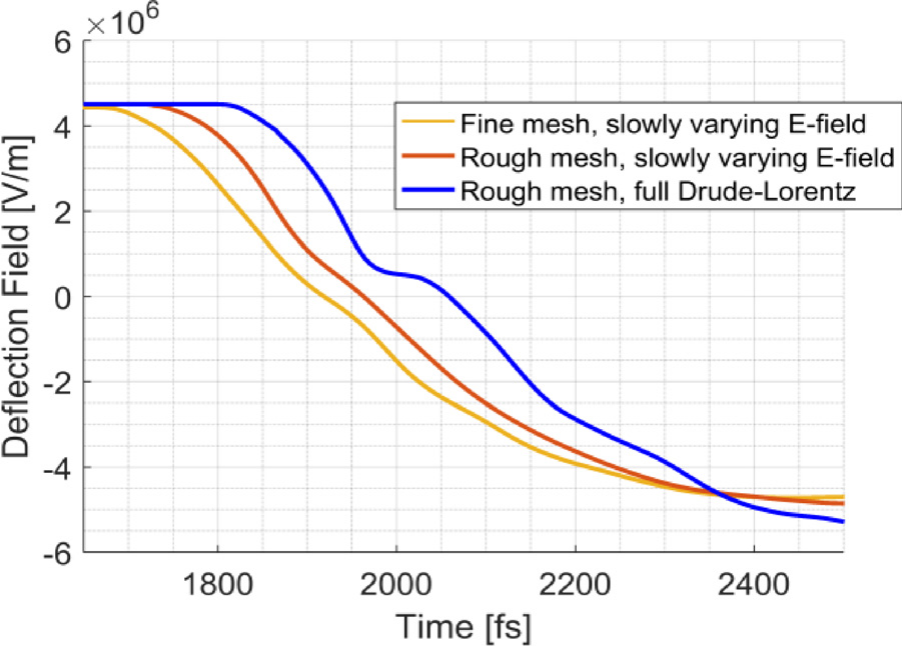
Deflection field vs time for the mesh and conductivity model as used in Fig. 4 (yellow line). A coarser mesh using the same conductivity model gives essentially the same time-dependence for the deflection field (red line). With the same coarse mesh and the Drude-Lorentz as described by Eq. (3), also a similar time-dependence is obtained (blue line).

The assumption of a relatively slowly varying electric field over the switch is valid for low laser powers because both models give a comparable result, as shown in Fig. 5. The time-dependent deflection field is shifted by about 100 fs for the full Drude-Lorentz model; however, this can be compensated by adjusting the path length of the laser pulse which is used to illuminate the photoconductive switch.

All electrodes are assumed to be perfect electrical conductors with zero thickness. Absorption and dispersion in the semiconductor are assumed to be negligible because the length of the deflector plate is small. The permittivity of GaAs varies only slightly from DC to terahertz frequencies, up to the point where the frequency approaches a phonon resonance, located around 8 THz.^19^

**FIG. 6.**
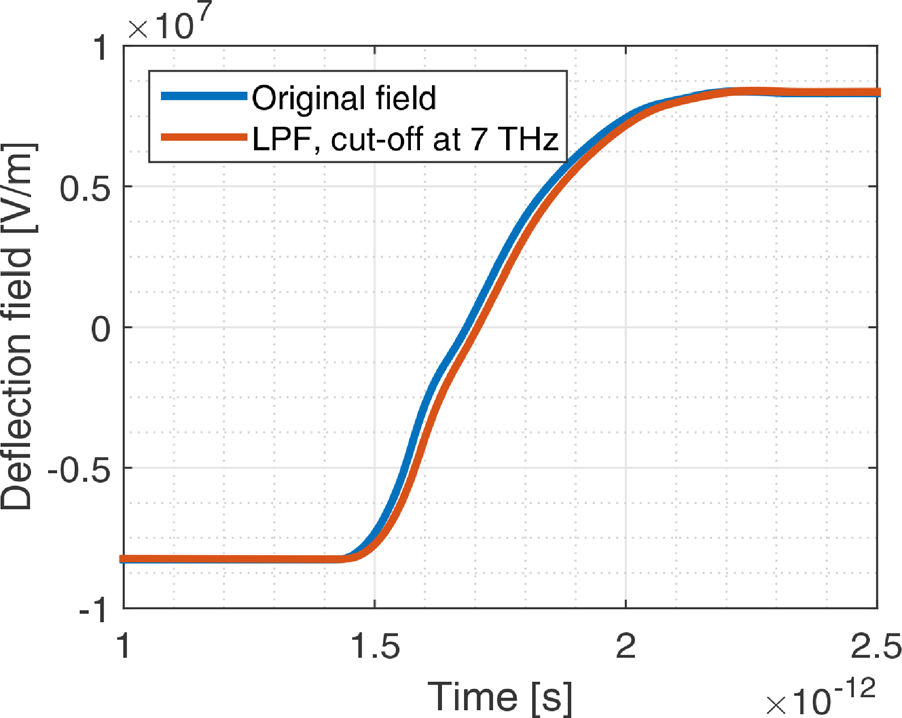
Deflection field as function of time. The blue curve is the original data and the red curve is the filtered data. The field is filtered with a first order Butterworth filter with a cut-off frequency of 7 THz to evaluate the influence of GaAs permittivity changes around the phonon resonance.

Around the phonon resonance, the real and imaginary parts of the permittivity vary significantly. To check the contribution of these frequencies to the deflection field, we filtered the data with a Butterworth first order low pass filter, shown in Fig. 6. We observe no change in the amount of time required to invert the deflection field in the deflector and, thus, conclude that it is reasonable to neglect the absorption due to the phonon resonance.

## PARTICLE TRACING OF THE ELECTRON BEAM AND BEAM QUALITY

In the section on “Model for the time-dependent conductivity of the photoconductive switch,” we calculated the time-dependent electromagnetic fields; in this section, we use this field to trace electrons through the blanker. This enables us to calculate the electron pulse length as well as potential blurring and energy spread increase in the beam.

The particle tracing module of COMSOL is used to calculate the trajectory of the electrons using Newton's second law. The Lorentz force acting on the electrons is calculated from the time-dependent magnetic and electric fields. Interaction between the particles and fields induced by the moving electrons is neglected.

**FIG. 7.**
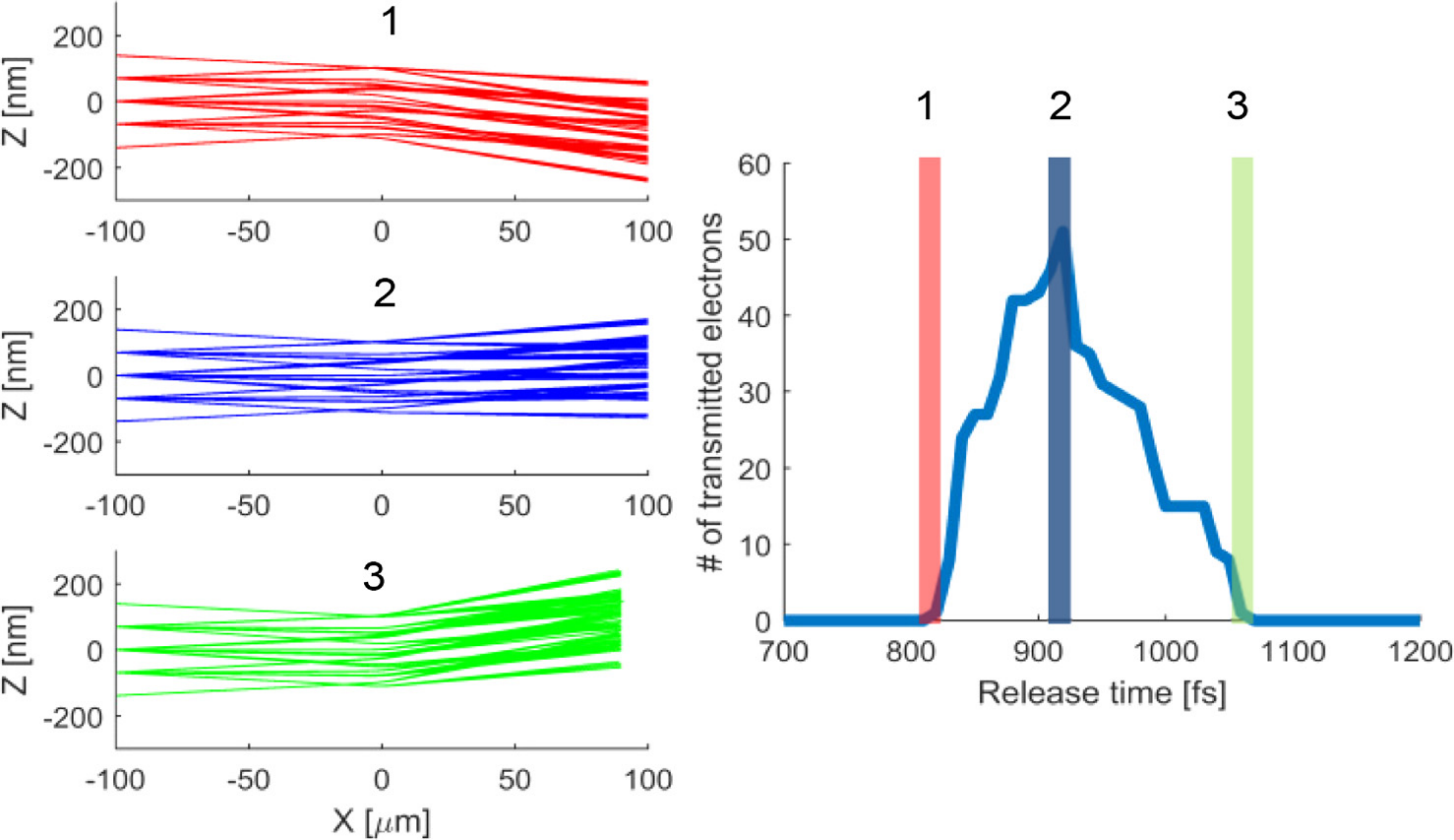
(1)–(3) Distance to the electron optical axis as function of position along the optical axis for release times of (1) 820 fs, (2) 960 fs, and (3) 1090 fs. The electrons are injected at x = −100 *μ*m with a velocity of 1 × 10^8^ m/s and are converging to a 200 nm crossover between the deflector plates located at x = 0 *μ*m. The colors of the rays indicate the different release times, in the right panel, which shows the amount of electrons transmitted through the blanker aperture as function of release time. An electron is transmitted through the aperture when the exit angle is larger than the half opening angle of the beam. In the simulations, a bunch of electrons is injected every 10 fs.

The FW50 spot size of a 4 nA electron beam will be 50 nm in the UFB for a realistic half opening angle of 0.4 mrad at 30 keV, and a 16 nA will have a FW50 spot size of 100 nm. A reduced brightness of 5 × 10^7^ A/(m^2 ^V^ ^sr^2^) is assumed, typical for a Schottky electron source, and an acceleration voltage of 28.5 kV. A Schottky source can have, and a cold field emitter does have, higher values for the reduced brightness as shown by van Veen *et al.*^26–28^ In the simulation, we want to include a broader part of the beam than only FW50, so we take a 200 nm focused electron beam spot in between the blanker plates with a half opening angle of 0.4 mrad. Every 10 fs, a bunch of 51 electrons is injected in the simulation to determine the amount blur induced by the deflector. In practice, a focused ultrafast electron pulse cannot have more than on average 0.5 electrons per pulse; otherwise, statistical electron interactions will reduce the brightness and increase the energy spread. The result of the particle tracing calculations is shown in Fig. 7.

**FIG. 8.**
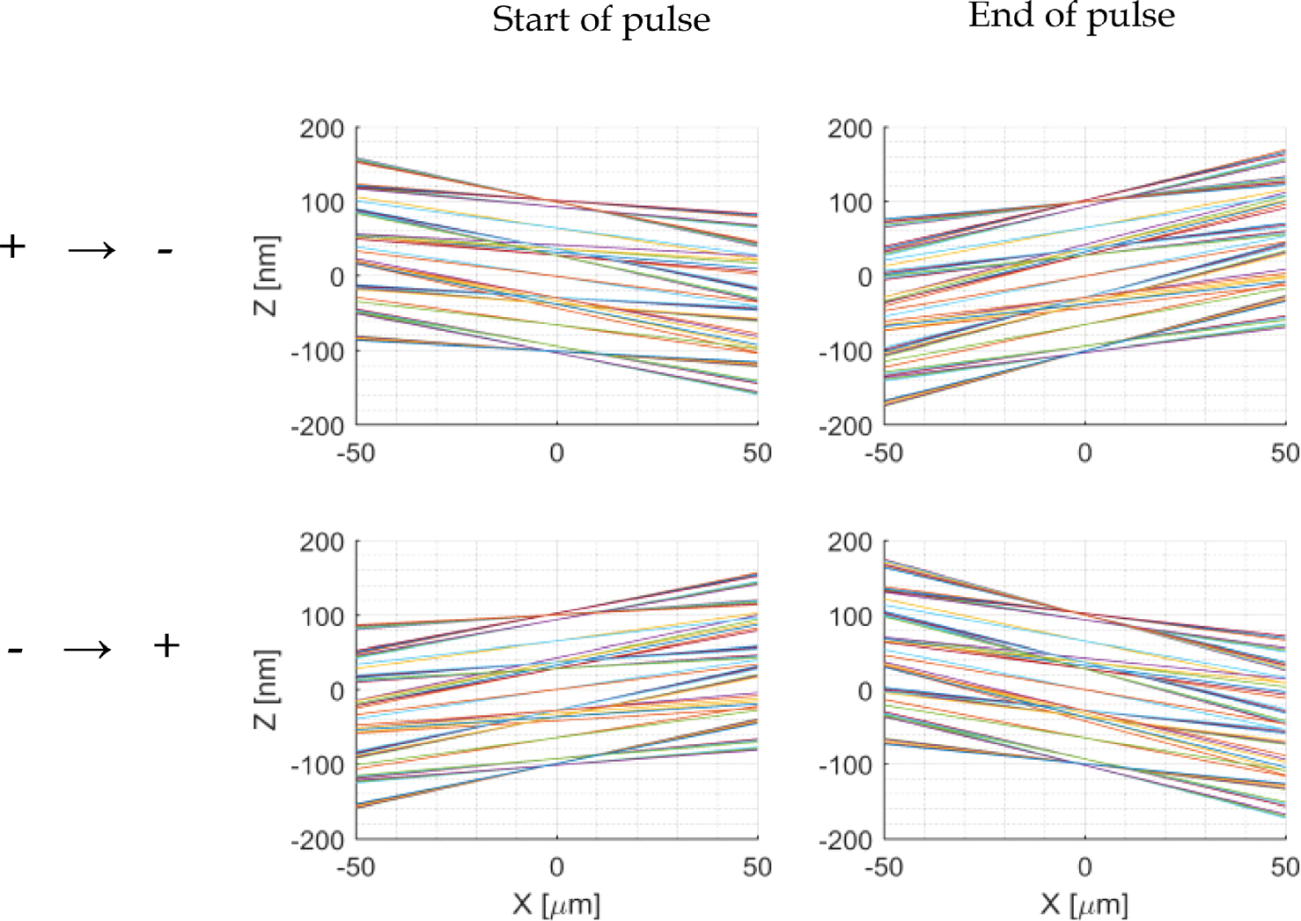
Distance to the electron optical axis as a function of position calculated by geometrically tracing back the electron trajectories. The top row shows the rays when the field sweeps from positive to negative and the bottom row shows the rays when the field sweeps from negative to positive. The left column shows the trajectories just before the first electrons are transmitted through the aperture and the right column the trajectories just after the last electrons transmitted through the aperture. The blanker is located at x = 0 *μ*m, the blur at all these positions is less than 5 nm.

The loss of brightness or blurring of the beam is analyzed by tracing the electron trajectories in a linear fashion back from the final position in the simulation to the beam blanker. We performed this analysis for two situations, one where the deflection field goes from positive to negative and the inverted case, see Fig. 8. The mesh size for this simulation is equal to “Simulation 1,” defined in the supplementary material information A. In all cases, a focus is created at the beam blanker with a beam displacement less than a couple of nanometers. This clearly shows that the increase in emittance can be neglected. We expect such a result for the reason that the residence time of electrons in the deflector is short, so the beam displacement is very small.

For each electron, the amount of deflection depends on the arrival time of the electron in the deflector with respect to the time-dependent electric field, the residence time of the electron in the deflector, and the rate of change of the field in the deflector. The net effect is that electrons in the front of the electron pulse have a different deflection angle when exciting the deflector than electrons in the back of the pulse. The net effect is a blur as the pulse seems to originate from different points in the conjugate image plan in the deflector. This blur is analytically estimated by calculating the equation of motion of an electron through the deflector and by considering the exponentially time varying field in the deflector as a simple linear ramp in field. The position and deflection of an electron in the back and front of a pulse is used to trace back where they appear to come from. This results in the following analytical equation, derived with the aid of Maple:

$$y=\frac{\mathrm{qE}L^{3}\tau_{e}}{24\phi \sqrt{\frac{2q\phi }{m}}\tau_{f}^{2}},$$ (8)

where *τ*_e_ is the electron pulse length, *τ*_f_ is the time constant of the exponentially rising field in the deflector, *E* is the maximum electric field, *ϕ* is the beam acceleration voltage, and *L* is the length over which the electron travels through the deflector. For the MEMS UFB, the blur will be equal to 0.3 nm, which will be a sub-angstrom contribution to the probe size in the sample plane and can be neglected.

## ENERGY GAIN INTRODUCED BY THE BLANKER

An electron approaching a static deflector will be accelerated and, when leaving the deflector, going to infinity, it will be decelerated back to its initial kinetic energy because the field is conservative. However, as in the UFB the voltage at the deflector is modulated in time, an electron can acquire a net energy gain or loss. The net energy gain or loss in the deflector, ΔE, is deterministically dependent on the position and injection time of the electron. The electron beam is monochromatic in the numerical calculation; hence, in reality the results should be convoluted with the 0.6 eV FW50 energy spread of a Schottky source or the 0.3 eV of a cold field emitter.^29^

**FIG. 9.**
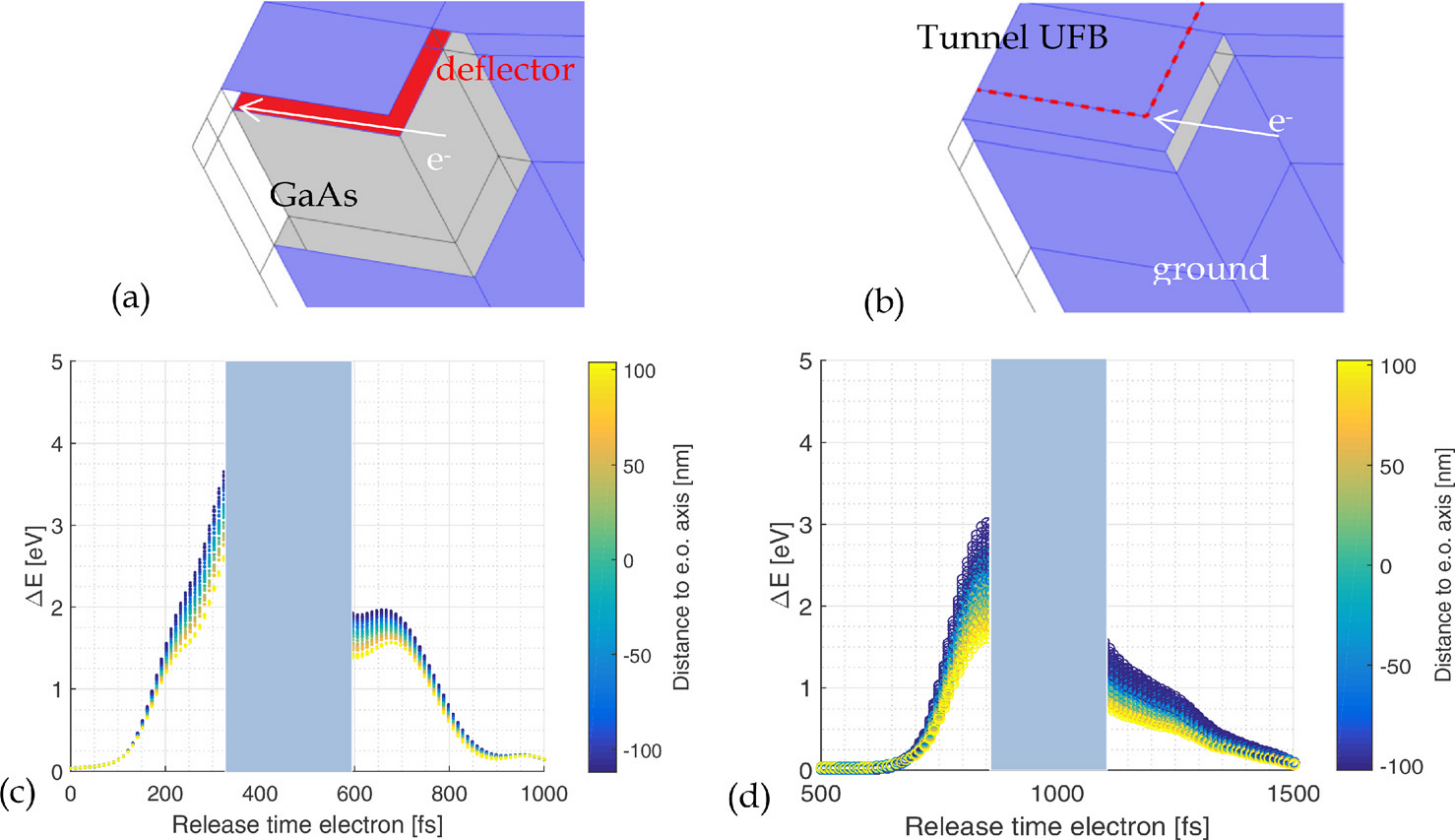
(a) Schematic indication of an open type design of the UFB. (b) Same for a tunnel-type design where fringe fields outside the blanker are shielded by the encapsulation. The red dashed line in the tunnel-type UFB indicates the contour of the deflector plate. (c) and (d) Energy gain of an electron as a function of release time in the simulation for (c) open design and (d) tunnel-type design. The color indicates the z position of the electron in the crossover; negative z values are closer to the deflector plate. The z-range of the electron trajectories varies from −100 nm to + 100 nm from the optical axis; the total distance between the deflector plates is 1 *μ*m. The voltage at the deflector plate inverted from +10 V to −10 V; hence, all electrons have some net energy gain. Electrons released between t = 310 fs and t = 570 fs are transmitted through the blanker aperture if it is located on the electron optical axis and accepts a half opening angle of 0.4 mrad; this region is marked in blue in the graph.

Figure 9(a) shows an “open” design of the UFB and Fig. 9(c) shows the energy gain of an electron traveling through such an open blanker as a function of both release time and position along the electron optical axis. The energy gain in this case is several electron volts. This relatively large energy gain is caused by the fringe fields around the deflector which increase the effective length of the deflector. We suppress this effect with a tunnel-type UFB where the deflector electrode is encapsulated with ground plates to reduce the spatial extent of the fringe fields [Fig. 9(b)]. The effect on the energy gain in the tunnel-type UFB is shown in Fig. 9(d). The tunnel-type UFB also has a lower potential at the optical axis, only 3.7 V, which further helps to reduce the energy gain.

As shown in Fig. 9, the energy gain is in the range of several electron volts. Electrons with the same release time also have energy dispersion, depending on their trajectory through the beam blanker. Trajectories closer to the deflector plate result in higher energy gain because the potential difference between entrance and exit of the deflector is higher. This effect can be minimized by reducing the probe size in the blanker. It also shows the importance of the (mechanical) stability of the UFB with respect to the electron optical axis. If the UFB is vibrating with an amplitude of about 100 nm, the energy spread of the electron beam will be noticeably increased. The same holds when the vibration amplitude of source multiplied with the magnification is on the order of 100 nm.

We note that electrons acquire on average ±4 eV energy for the open UFB design, with the sign depending on whether the voltage flips from positive to negative or the inverse. Hence, due to chromatic aberrations of the lens, two spots will be visible in the image plane. For a final lens with a *C_c_* = 10 mm and a half opening angle of 5 mrad, the two spots are separated by about 14 nm at 28.5 kV. An additional blanker might be used to pick up only the even or odd pulses in order to mitigate this effect. The effect could be further reduced by making a new design in which both deflector plates would be excited by opposite voltage pulses.

## ESTIMATION ENERGY GAIN OF THE UFB

For a better understanding, we will discuss analytical models to approximate the energy gain. This is useful because it allows us to minimize the energy spread for a certain required peak current and electron pulse length.

The deflection of the electron beam is quite small and the energy gain is negligible compared to the total energy of the electron beam; hence, we can assume a constant velocity and zero beam displacement. For this reason, the energy gain can be estimated as follows:

$$\Delta E\left(t_{0}\right)=q\int_{-\infty }^{+\infty }E_{x}(x,t_{0}+x/v)\mathrm{dx},$$ (9)

where *E*_x_(*x*, *t*) is the electric field component along the electron optical axis as a function of time and position, *v* is the velocity, and *t*_0_ is the release time of the electron in the simulation.

As a first estimation of the energy gain, quasi-static electric fields are assumed. The term “quasi-static” implies a potential that can be described as a product of a spatial function, *ϕ*(**x**), and a temporal one, *f*(*t*),

ϕ(x,t)=ϕ(x)f(t). (10)

**FIG. 10.**
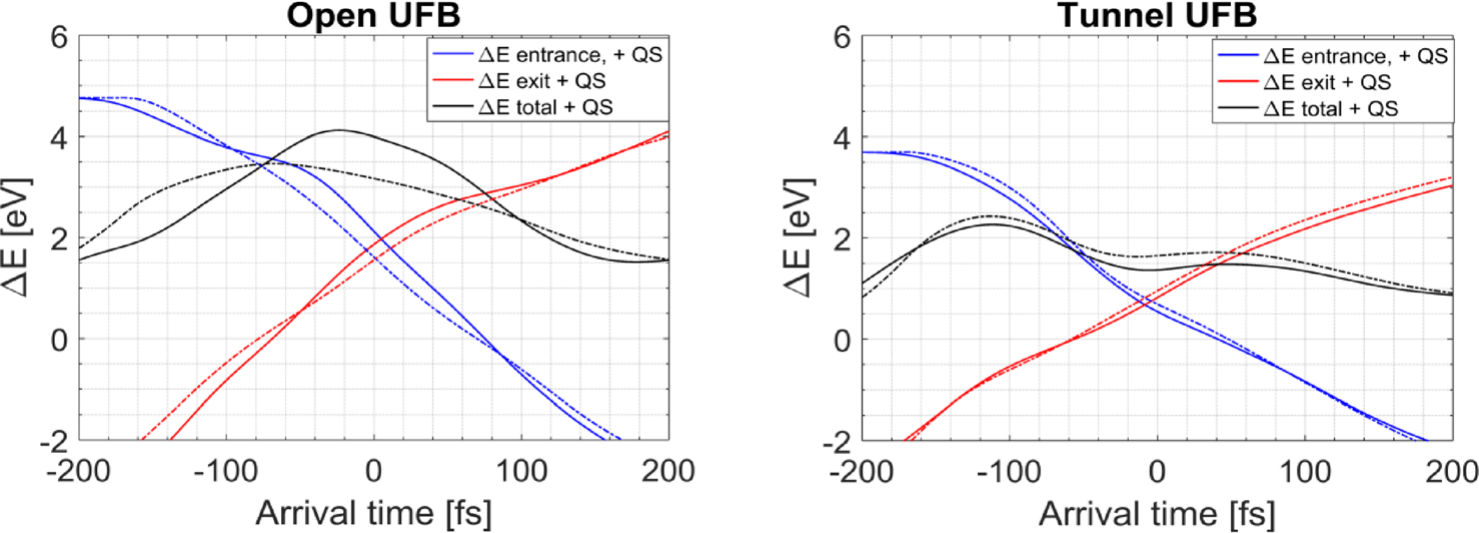
Energy gain as a function of electron arrival time in the UFB for (left panel) the open design and (right panel) the tunnel-type design. Solid lines indicate the total energy gain (black line), the energy gain when entering the deflector (blue line), and when leaving the deflector (red line). The dashed lines are the calculated energy gains when the fields are approximated as being quasi-static. (left) Calculated energy gain for the open design and (right) calculated energy gain for the tunnel design.

The quasi-static approximation is checked using our simulation data of the fields in combination with Eq. (9). For the spatial distribution *ϕ*(**x**), we use the initial potential, *f*(*t*) is approximated by using the normalized deflection field in the center of the deflector. With this method, the quasi-static energy gain as a function of arrival time is calculated and compared with the energy gain calculated with Eq. (9) and using the full time-dependent fields. This enables us to check whether approximation of the fields as quasi-static is valid or not. The result is shown in Fig. 10. For the tunnel UFB design, the quasi-static approach provides an adequate approximation with a relative error smaller than 20%, while for the open UFB design, the deviation with the quasi-static approach can be substantial, up to a few electronvolts. The tunnel-type design limits the spatial extent of the fringe fields, so retardation effects in the potential are suppressed.

**FIG. 11.**
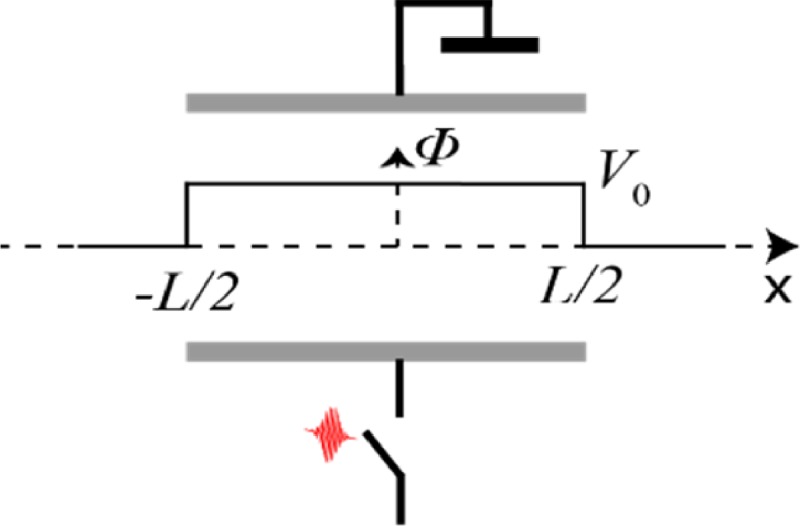
Potential, ϕ, along the electron optical axis, x. The potential has a square shape with an amplitude equal to V_0_; the deflector has a length equal to L.

The energy gain is a function of arrival time and we are interested in an analytical expression for the energy gain. For the analytical approximation of the energy gain, a description of the potential *ϕ*(*x*) and the time-dependent function *f*(*t*) is required. The potential along the electron-optical axis is approximated with a square potential, see Fig. 11.

The time-dependent function is described with a polynomial

$$f\left(t+\tau \right)=\alpha_{1}\left(\frac{x}{v}+\tau \right)+\alpha_{2}{\left(\frac{x}{v}+\tau \right)}^{2}+\alpha_{3}{\left(\frac{x}{v}+\tau \right)}^{3}+\cdots ,$$ (11)

where *τ* is the arrival time of the electron in the deflector. Note that *f*(*t*) is a dimensionless function, so the parameters *α*_1_, *α*_2_, etc., have units of [s^−1^], [s^−2^], etc. Zeroth order terms are taken out of the polynomial because they do not affect the final energy. We calculated the energy gain for the different terms in the polynomial using Eq. (11) (see supplementary material information B)

$$\begin{array}{c} \Delta E_{1}\left(\tau \right)=V_{0}\alpha_{1}\frac{L}{v},\\ \Delta E_{2}\left(\tau \right)=2V_{0}\alpha_{2}\frac{L\tau }{v},\\ \Delta E_{3}\left(\tau \right)=V_{0}\alpha_{3}\left(\frac{1}{4}\frac{L^{3}}{v^{3}}+\frac{3L\tau^{2}}{v}\right). \end{array}$$ (12)

The first term describes the fact that all electrons either gain or lose a constant amount of energy, depending on the sign of *α*_1_. The second term describes that the blanker is either compressing or expanding the electrons pulse, like a buncher. Whether the beam blanker expands or compresses the pulse depends on the sign of *α*_2_. This effect is known from the literature; Thong discussed that a parallel plate blanker acts as a buncher and that the bunching effect can be reduced substantially by using symmetrical fields^12^ as in that case the potential is zero along the electron optical axis. Third order effects introduce a combination of a constant energy difference and a quadratic energy change as a function of the arrival time in the beam blanker.

All ultrafast beam blankers, to our knowledge, currently for use in UEM use sinusoidal fields. Sinusoidal fields have the advantage that they can easily be amplified with resonant structures, necessary to generate high slew rate deflection fields resulting in short electron pulses. The UFB presented here is different because the deflection field has a broad spectrum in the frequency domain. We generate high slew rate deflection fields, by deflecting the beam with fields in the terahertz frequency domain instead of at gigahertz frequencies. A fundamental difference between both approaches is that the second order term for the energy gain is zero when sinusoidal fields are used, as shown in Table I.

**TABLE I.** Expressions for the expansion terms in the time-dependent part of the blanking potential in quasi-static approximation for both sinusoidal and exponential deflection fields.

	First order	Second order	Third order
Sinusoidal field: $V\left(t\right)=V_{0}\;\text{sin}\;\left(2\pi t/\tau_{f}\right)$	$\alpha_{1}=2\pi /\tau_{f}$	$\alpha_{2}=0$	$\alpha_{3}=8\pi^{3}/6\tau_{f}^{3}$
Exponential field: $V\left(t\right)=V_{0}\left(1-2\;\text{exp}\;\left(-t/\tau_{f}\right)\right)$	$\alpha_{1}=2/\tau_{f}$	$\alpha_{2}=-1/\tau_{f}^{2}$	$\alpha_{3}=1/3\tau_{f}^{3}$

With the terms in Table I and using reduced brightness, *B*_r_, current, and FW50 electron pulse length Δ*t*, the expressions for the energy gain can be rewritten with the aid of the Maple software, to find (see supplementary material information B)

$$\begin{array}{c} \Delta E_{1}=4\sqrt{2}\frac{V_{0}\sqrt{I}}{E_{0}\Delta td_{p}\pi \sqrt{q/m}\sqrt{B_{r}}},\\ \Delta E_{2}\left(\tau \right)=-16\sqrt{2}\frac{IV_{0}\sqrt{\phi }}{{\left(\pi d_{p}E_{0}\Delta t\right)}^{2}\sqrt{q/m}LB_{r}}\tau , \end{array}$$ (13)

where *E*_0_ is the initial strength of deflection field in the UFB, *d_p_,* is the FW50 probe size, and *I* is the DC beam current in the UFB. The equations clearly show the importance of a small distance between the deflector plates, *d*: The first order term increases linearly with *d* and the second order quadratically. A high reduced brightness source is also essential for the UFB; it allows more current in the pulse for a given energy gain.

**FIG. 12.**
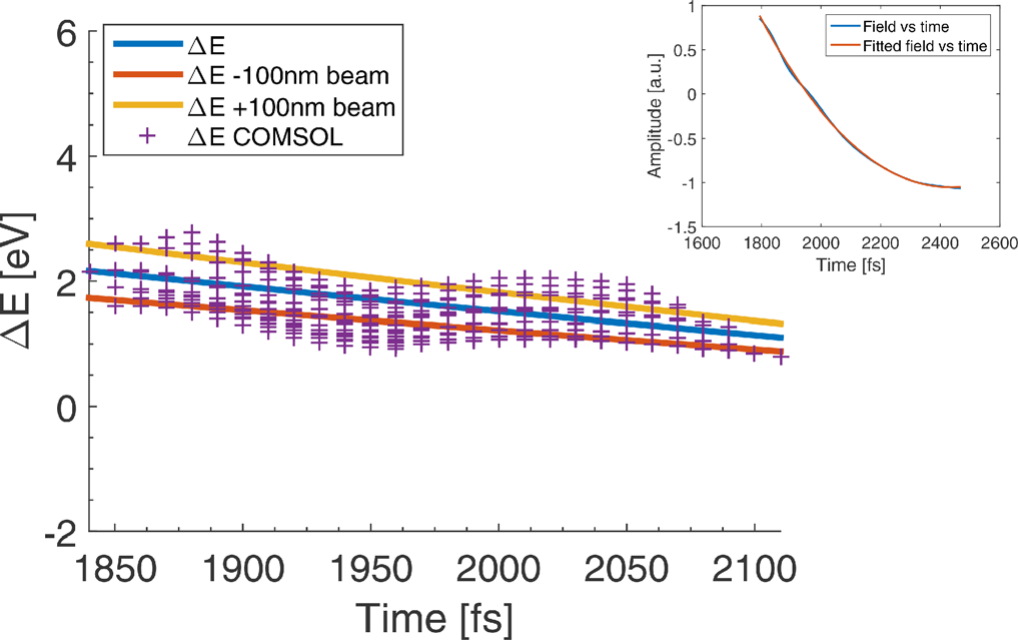
Energy gain as function of arrival time in the tunnel-like deflector calculated with Comsol (purple crosses).The blue line is the energy gain as a function of time for a beam propagating along the electron optical axis calculated with Eq. (12). (inset) Result of the polynomial fit to fit the deflection field in the deflector. The yellow and red lines describe the energy gain of the 100 nm off-axis beams.

The approximations for the energy gain are compared with the numerical simulation. The normalized deflection field is fitted to calculate the *α*-coefficients in the polynomial (see the inset in Fig. 12), which yields values of −4.47 × 10^12^ s^−1^, 5.34 × 10^24^ s^−2^ and −8.39 × 10^35^ s^−3^ for *α*_1_, *α*_2_, and *α*_3_, respectively. Subsequently Eq. (12) is used to calculate the energy gain as function of arrival time in the blanker. The result of this calculation is shown in Fig. 12.

With the simplified quasi-static model and a square-like potential, a reasonable estimation of the energy gain for a tunnel-type UFB is obtained. Only the small oscillations in the energy gain as a function of arrival time are not described with the approximations we use. Using a similar approximation of the energy gain around the open-type UFB will not work due to the significant effect of nonquasi-static fields on the energy gain.

## ESTIMATION ENERGY SPREAD

As a result of the time- and trajectory-dependent energy gain in the blanker that we numerically calculated in the section on “Estimation energy gain of the UFB,” the energy spread in the electron beam will change. In the remainder of the section, we will refer to this as the energy spread introduced by the UFB. A second, less important, parameter is the first order constant energy gain, ΔE_1_, introduced by the UFB because its value determines whether an additional blanker is required to intercept the even or odd electron pulses. It is important to know the range of beam currents and electron pulse lengths in which the energy spread and constant energy gain are low.

**FIG. 13.**
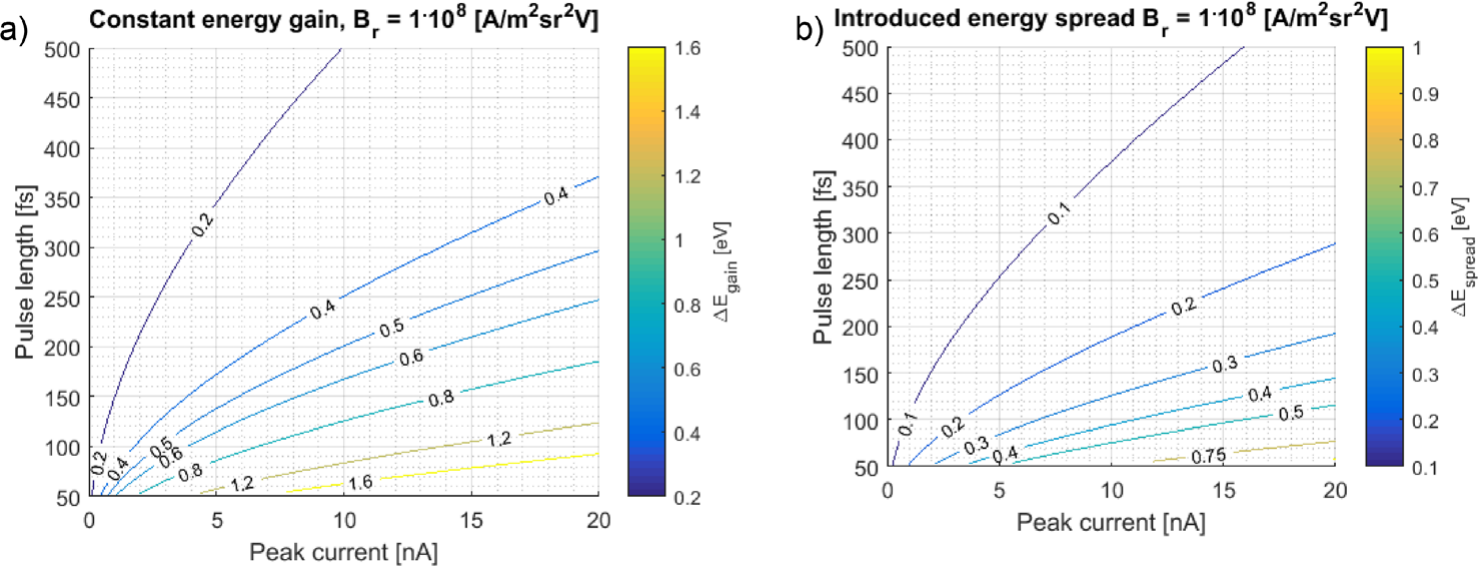
(a) Constant energy gain as a function of current and FW50 pulse length at 28.5 keV beam energy. For low currents and long electron pulses, the energy gain is small compared to the source energy spread and both even and odd electron pulses can be used. (b) Energy spread in the electron pulse as a function of current and pulse length. In both figures, we assume a probe size of 175 nm in the UFB. The peak current is the current in the electron pulse which is equivalent to the average beam current between the blanker plates. The Comsol energy spread calculation presented in Fig. 12 corresponds to a pulse length of 125 fs and a current of 16 nA.

In Fig. 13(a), the constant energy gain is plotted for a range of pulse lengths and beam currents, as calculated using the simplified quasi-static approximations. This clearly shows that for electron pulse length longer than 400 fs and current below 5 nA both even and odd pulses from the blanker can be used as there is a 0.4 eV energy difference between the even and odd pulses.

The calculated energy spread is shown in Fig. 13(b) for the situation where the separation between the blanker plates is 1 *μ*m. The energy spread introduced by the UFB is estimated by the squared addition of the difference in off-axis energy gain over the FW50 spot size, the difference in second order energy gain over the FW50 pulse length, and the third order effect. The latter has a very small contribution, lower than 0.025 eV. The energy spread depends on the size of the electron spot in the UFB, for which we took a value of 175 nm. The energy spread can be further reduced by increasing the probe size; however, the larger the probe size, the more electrons hit the sides of the UFB. The spot in the UFB is a magnified image of the virtual source which has a certain shape, for example a Gaussian. A Gaussian probe of 175 nm FWHM will have a 10^−4^ fraction in the tails that extend into the deflector plates. It is unknown how many of these electrons scatter such that they end up in the sample chamber. However, if only a small fraction of the scattered electrons ends up in the sample chamber, the resulting background signal may still be significant and visible in a pump-probe measurement as the duty cycle of the blanker is only 10^−5^.

## TEMPORAL DISTORTION OF THE ELECTRON PULSE DUE TO ENERGY SPREAD AND MAGNETIC LENS

An electron pulse traveling through free space over a distance *L_s_* at an energy *ϕ* will be delayed by time −Δ*t* if it acquires an additional energy *δE*. This Δ*t* is described by

$$\Delta t=L_{s}\left(\frac{1}{v+\Delta v}-\frac{1}{v}\right)=-L_{s}\frac{\Delta v}{v^{2}+v\Delta v}\approx -L_{s}\frac{\Delta v}{v^{2}}=-L_{s}\frac{\delta E}{2\sqrt{\frac{2e}{m}}\phi^{3/2}}.$$ (14)

The equation shows that there is an approximate linear relationship between the energy loss and the broadening of the electron pulse. At a beam energy of 30 keV, the dispersion is 160 fs/eV m; hence a 1 eV energy difference over a drift space of 0.2 m will induce a pulse broadening of 32 fs. This 0.2 m is a typical value for the distance between the UFB and the sample. Thus, the beam blanker cannot generate electron pulses shorter than about 20 fs, provided that the source has an energy spread of 0.6 eV. Note that the electron source is even further away from the sample, so this effect will be stronger when the pulses are created by direct laser irradiation of the source.

**FIG. 14.**
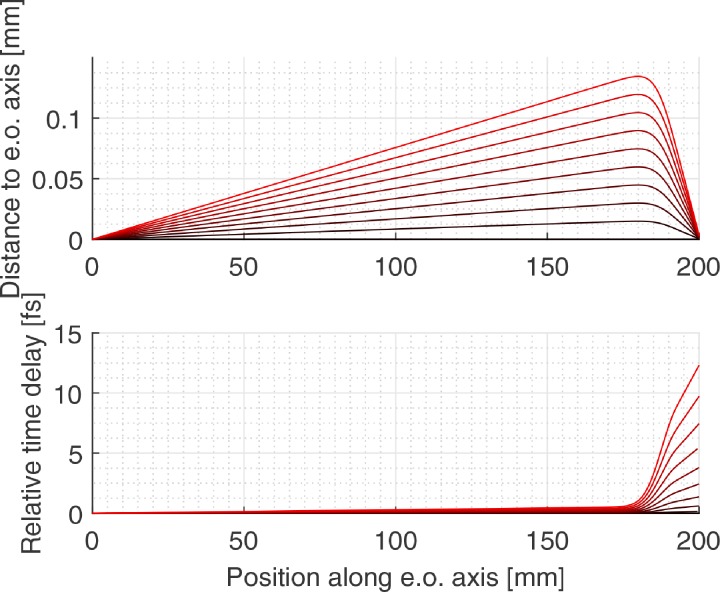
Temporal distortion of the electron pulse induced by the magnetic lens in a typical SEM system. The position of the UFB is at *x* = 0, the beam energy is 30 keV, and the crossover in the UFB is demagnified on the sample by means of a magnetic lens, calculated with EOD. The most outer ray in the graph corresponds to a half opening angle of 0.8 mrad, where 0.4 mrad is a typical working value. Thus, the temporal distortion induced by the magnetic lens is typically less than 5 fs.

The final lens of the microscope will also induce a broadening of the pulse as discussed by Weninger and Baum.^30^ They calculated the temporal distortion for an electron pulse originating from a photocathode and focused by a magnetic lens. We calculated the temporal distortion of an electron pulse originating from a crossover located before the final magnetic lens as shown in Fig. 14, the temporal distortion is small, less than 5 fs for typical opening angles.

The combination of the energy spread of the source, the energy spread induced by the UFB, and the temporal distortion implies that the electron pulse lengths will be increased by roughly 30 fs.

## INFLUENCE OF EVEN VS ODD UFB VOLTAGE SWITCHING

In our concept of an UFB, the deflector electrode inverts from positive to negative voltage for even pulses and oppositely for uneven laser pulses.^1,10^ For an even laser pulse, the electrons in the pulse will encounter a net energy gain. However for uneven pulses, there will be a net energy loss. For the even pulses, the deflector is at the anode side of the switch and for the uneven pulses at the cathode side; hence, a difference in the electron pulse length is expected. For electron pulses of a few 100 fs and shorter, an additional deflector would be required to intercept the odd pulses to reduce the effective energy spread and obtain shorter electron pulses.

In all our simulations, we put the deflector plate at the anode side of the photoconductive switch. We expect a significant difference in performance whether the deflector is at the anode or cathode side of the photoconductive switch. Higher amounts of terahertz radiation are generated when the laser spot illuminates the switch at locations closer to the anode side because the mobility at the anode is higher than at the cathode.^31–33^ At the anode, electrons in the GaAs layer will be taken up quickly due to their low effective mass, while this process will be slower at the cathode. Hence, when the deflector plate is at the anode, it will be quickly decharged because electrons are injected at a high rate into the deflector plate.

## CONCLUSIONS

A full time-dependent electromagnetic FEM simulation of a photoconductive switch integrated with a MEMS-sized deflector is performed in combination with particle tracing of an electron beam to study the dynamics of the resulting pulsed electron beam. The numerical calculations show that it is possible to invert the deflection field on a time scale of about 500 fs. This value is comparable with measured THz pulse lengths created with LT-GaAs photoconductive switches illuminated with femtosecond laser pulses.^20,34^

The particle tracing simulations show a negligible increase in emittance, demonstrating that the brightness of the electron pulse will remain high. Depending on the chosen opening angle of the electron beam, electron pulse lengths shorter than 150 fs can be achieved.

The numerical calculations further show that, in case the fringe fields extend far enough outside the UFB, the effective increase in deflector length with retardation effects of the EM fields outside the deflector has a significant effect on the energy gain of an electron after propagating through the deflector. Analytical equations of the time-dependent fields extending outside the deflector are hard to derive; hence, numerical calculations are required to calculate the energy gain. In a tunnel-type design where the fringe fields are confined by shielding the deflector electrode with conducting grounded plates, the energy spread in the pulse will be limited to 0.5 eV. This is comparable to or lower than the energy spread of a Schottky electron source. In such a case, the induced energy gain can be quite accurately estimated, provided that it is known how fast the deflection field inverts. The encapsulation of the deflector electrode in the tunnel-type design reduces the energy spread for two reasons. The first one is that it limits the extent of the fringe field and, hence, the effective length of the deflector. The second reason is that because of the more limited spatial extent of the potential outside the deflector, retardation effects in the potential can be neglected.

Finally, we argued that the further temporal distortion of the electron pulse by the final (magnetic) lens in a SEM is less than 10 fs and that temporal broadening of the electron pulse between the UFB and sample is about 30 fs. Thus, we expect that electron pulses of about 100 fs are achievable using a laser-triggered beam blanker in a SEM, with a negligible increase in emittance and with only a marginal increase in energy spread.

## SUPPLEMENTARY MATERIAL

See supplementary material for graphs with the sensitivity of the numerical calculations to changes in mesh size. A detailed mathematical derivation is given for the first and second order energy gain introduced; the beam deflector is also described. The supplementary material also contains a MAPLE script to determine the amount of beam blur introduced by the ultrafast beam blanker.
